# Determination and Analysis of Ustiloxins A and B by LC-ESI-MS and HPLC in False Smut Balls of Rice

**DOI:** 10.3390/ijms130911275

**Published:** 2012-09-10

**Authors:** Tijiang Shan, Weibo Sun, Hao Liu, Shan Gao, Shiqiong Lu, Mingan Wang, Wenxian Sun, Zhiyi Chen, Shu Wang, Ligang Zhou

**Affiliations:** 1 College of Agronomy and Biotechnology, China Agricultural University, Beijing 100193, China; E-Mails: shan5400388@163.com (T.S.); sunweibo.1001@163.com (W.S.); hardyliuhao@163.com (H.L.); gaoshankonglong@163.com (S.G.); shiqionglu@126.com (S.L.); wangma@cau.edu.cn (M.W.); wxs@cau.edu.cn (W.S.); 2 Institute of Plant Protection, Jiangsu Academy of Agricultural Sciences, Nanjing 210014, China; E-Mail: chzy@jaas.ac.cn; 3 Institute of Plant Protection, Liaoning Academy of Agricultural Sciences, Shenyang 110161, China; E-Mail: wangshu509@163.com

**Keywords:** *Villosiclava virens*, ustiloxins A and B, false smut balls of rice, determination and analysis, LC-ESI-MS, HPLC

## Abstract

Ustiloxins are cyclopeptide mycotoxins produced by the pathogenic fungus *Villosiclava virens* of rice false smut. Ustiloxins A and B as two main mycotoxins were determined conveniently by LC-ESI-MS in the water extract from rice false smut balls which were mostly composed of the chlamydospores and mycelia of the pathogen. Both ustiloxins A and B in the water extract were also quantitatively analyzed by HPLC. This is the first report on the determination and analysis of ustiloxins A and B simultaneously by LC-ESI-MS and HPLC in false smut balls of rice.

## 1. Introduction

Ustiloxins are cyclopeptide mycotoxins containing a 13-membered cyclic core structure produced by the pathogenic fungus *Villosiclava virens* (Nakata) Tanaka & Tanaka (anamorph: *Ustilaginoidea virens* Takahashi) [1–4] of rice false smut which is an emerging and increasingly significant disease in most rice (*Oryza sativa* L.) producing countries (*i.e*., China, India, Burma, and Japan) [5,6]. Ustiloxins are toxic to plants and animals especially because their cytotoxic activity is antimitotic by inhibition of microtubule assembly and cell skeleton formation [7]. Nakamura *et al*. reported that both ustiloxin A and the crude fraction obtained from the water extract of rice false smut balls caused liver and kidney damage in mice [8]. These findings indicate that both false smut balls and false smut pathogen-infected rice food and forage create concerns for food and feed safety. An assessment of the risk to animals and humans, and the establishment of assay methods for the toxic substances in the false smut balls are needed before rice food or forage can be utilized effectively. In order to detect ustiloxins in samples, only one report has been published on ustiloxin A analysis by HPLC in forage rice [9]. The purpose of this investigation was to seek practical methods for isolating, analyzing and determining the main ustiloxins in a variety of samples (*i.e*., false smut balls, mycelia, grains, forage rice, and their products). In this study, two main ustiloxins (*i.e.*, ustiloxins A and B) were isolated from rice false smut balls. A high-performance liquid chromatography-mass spectrometry (HPLC-MS) method for rapidly detecting ustiloxins A and B, as well as a high-performance liquid chromatography (HPLC-UV) method for quantitatively analyzing the content of ustiloxins A and B in rice false smut balls were then established.

## 2. Results and Discussion

### 2.1. Primary Determination of the Ustiloxin-Like Compounds in Rice False Smut Balls by LC-ESI-MS

**Figure 1:**
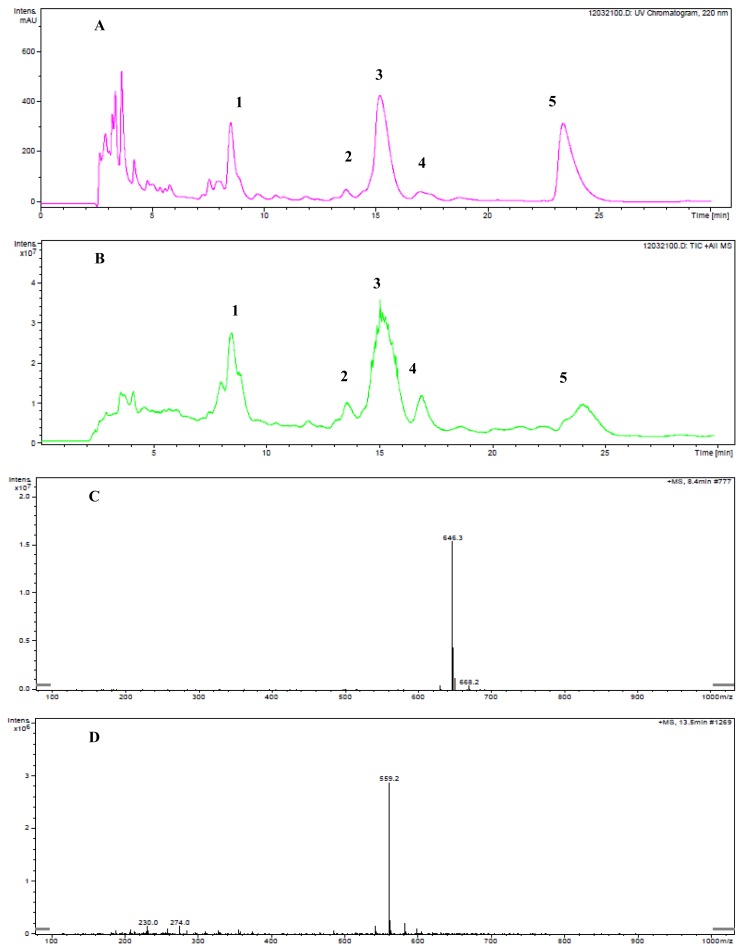

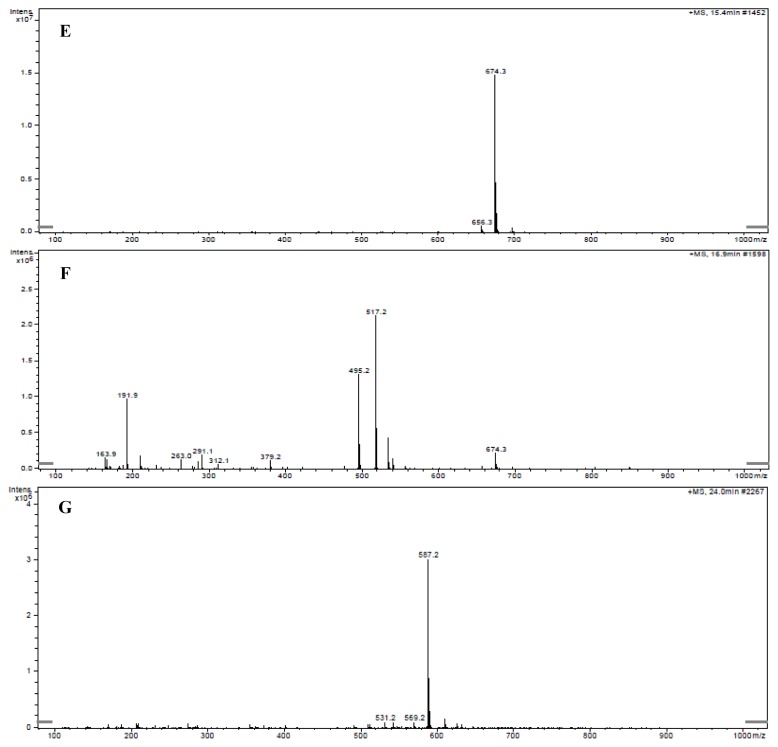
Primary determination of the ustiloxin-like compounds from the water extract of rice false smut balls by LC-ESI-MS. (**A**) and (**B**) were the spectra of HPLC-UV and total ion chromatograms, respectively. (**C**–**G**) were the ESI-MS spectra of peaks 1, 2, 3, 4 and 5, respectively, appearing in (**A**) and (**B**).

Figure 1 shows the primary determination of the ustiloxin-like compounds from the water extract of rice false smut balls by LC-ESI-MS. The spectrum with detection wavelength from 190 to 400 nm was employed to obtain more peaks in the HPLC-UV chromatogram. The major detection wavelength of 220 nm was selected. Five peaks (*i.e*., peaks 1–5) appeared in both HPLC-UV and total ion chromatograms (Figure 1A,B) with retention times of 8.4 min, 13.5 min, 15.4 min, 16.9 min and 24.0 min, respectively. They were selected for further ESI-MS analysis with the results shown in Figure 1. By comparison with the molecular weights of the ustiloxins previously reported [1,10,11], we preliminarily concluded that peaks 1–4 should be ustiloxin-like compounds. At 646.3 *m/z* (Figure 1C) should be the peak of the [M + H]^+^ ion of ustiloxin B that corresponds to peak 1 in Figure 1A,B. Similarly, at 559.2 *m/z* (Figure 1D) should be the peak of the [M + H]^+^ ion of ustiloxin C that corresponds to peak at 2, 674.3 *m/z* (Figure 1E) should be the peak of the [M + H]^+^ ion of ustiloxin A that corresponds to peak 3, and at 495.2 *m/z* and 517.2 *m/z* (Figure 1F) should be the peaks of the [M + H]^+^ and the [M + Na]^+^ ions of ustiloxin D that correspond to peak 4 in Figure 1A,B. These ustiloxin-like compounds need to be purified and further confirmed by spectroscopic methods such as NMR and MS. According to the peak areas in the HPLC profile (Figure 1A) and the ion relative abundances in the TIC spectrum (Figure 1B), peaks 1, 3 and 5 should correspond to the three main components in the water extract of rice false smut balls. Peak 5 (in Figure 1A,B) with a major ion peak at *m/z* 587.2 shown in Figure 1G has not been reported previously. It needs to be purified and further confirmed.

### 2.2. Elucidation of the Purified Ustiloxins A and B

**Figure 2:**
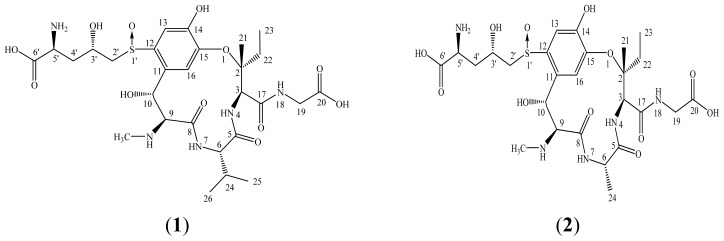
Chemical structures of the ustiloxins A (**1**) and B (**2**).

After repeated column chromatographic purification on macroporous adsorption resin HP-20, dialysis, GH gel ODS-AQ, Sephadex LH-20, and Sephadex Q15, the water extract of rice false smut balls afforded two compounds (**1** and **2**). After comparing their physicochemical and spectrometric data with those reported in the literature, they were identified as ustiloxins A (**1**) [10], and B (**2**) [1,11], respectively, whose structures are shown in Figure 2.

### 2.3. Determination of Ustiloxins A and B by LC-ESI-MS

**Figure 3:**
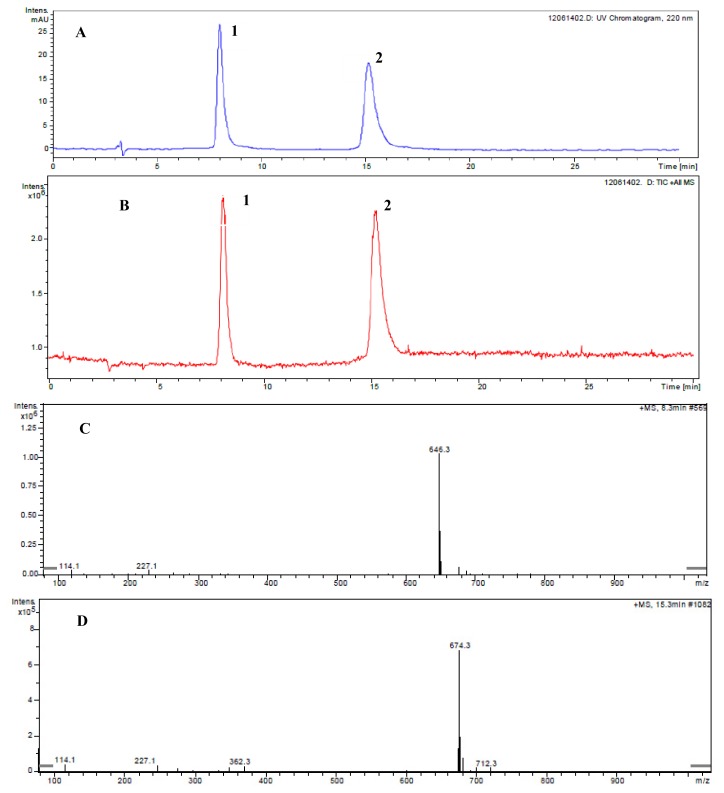
Determination of the ustiloxins A and B by LC-ESI-MS. (**A**) and (**B**) show the spectra of HPLC-UV and total ion chromatograms, respectively. (**C**) and (**D**) show the ESI-MS spectra of ustiloxins B (peak 1) and A (peak 2), respectively, appearing in Figures 1A,B.

With the same conditions as used in Figure 1, both the purified ustiloxins A and B were mixed for LC-ESI-MS analysis. Figure 3 shows the determination of ustiloxins A and B by LC-ESI-MS. At 646.3 *m/z* (Figure 3C) was the peak of the [M + H]^+^ ion of ustiloxin B that corresponds to peak 1, and at 674.3 *m/z* (Figure 3D) was the peak of the [M + H]^+^ ion of ustiloxin A that corresponds to peak 2 in Figure 3A,B. The retention times of ustiloxin B (peak 1) and ustiloxin A (peak 2) in Figure 3A,B were 8.3 min and 15.3 min, respectively, which were almost the same as peaks 1 and 3 in Figures 1A,B. The results confirmed that ustiloxins A and B as the two main ustiloxins in rice false smut balls can be determined by an LC-ESI-MS method.

### 2.4. HPLC Analysis of Ustiloxins A and B in Rice False Smut Balls

#### 2.4.1. Identification of Ustiloxins A and B by HPLC-UV

**Figure 4:**
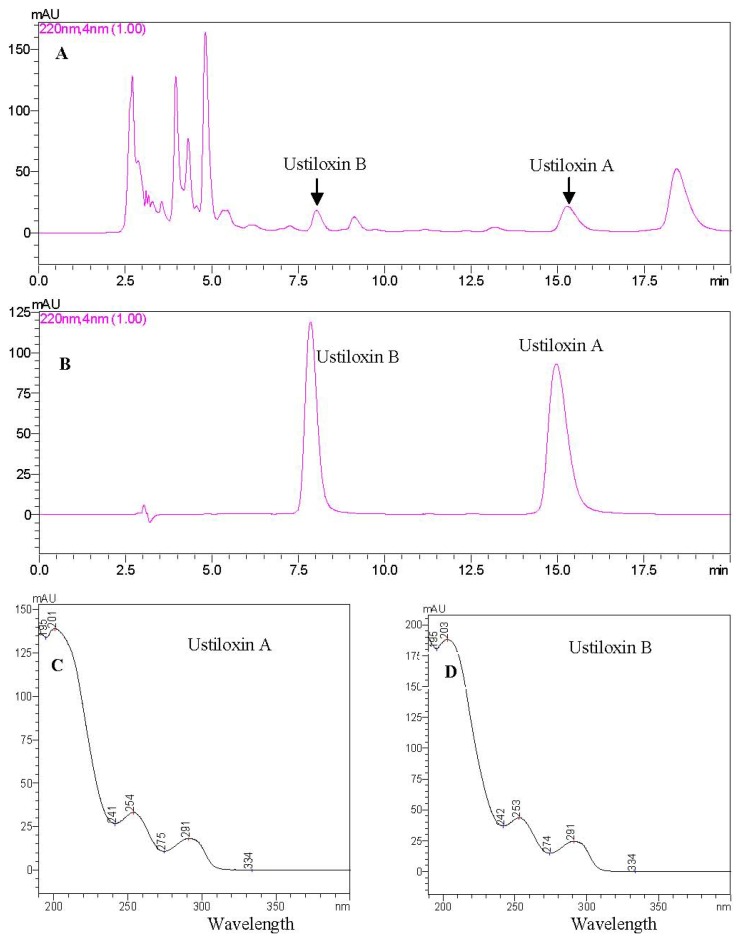
HPLC-UV analysis of ustiloxins A and B in rice false smut balls. (**A**) is the HPLC profile of the water extract; (**B**) is the HPLC profile of the reference ustiloxins A and B. Figures 4**C** and 4**D** are the UV absorption spectra of ustiloxins A and B, respectively.

A rapid HPLC method was developed for analysis of ustiloxins A and B in rice false smut balls. Figure 4A,B show the HPLC-UV profiles of the water extract and reference ustiloxins A and B, respectively. Both ustiloxins A and B in rice false smut balls were identified by comparison of their retention times with the reference ustiloxins as well as their UV absorption spectra (Figure 4C for ustiloxin A, and Figure 4D for ustiloxin B). HPLC analysis was complete in 20 min.

#### 2.4.2. Calibration Curves

Based on the above results, ustiloxins A and B were selected for quantitative analysis by HPLC. The linear equation for ustiloxin A analysis was *Y =* 2969445.7810*X −* 55531.7034 (*R* = 0.9998), and that for ustiloxin B was *Y =* 2394672.3039*X −* 79066.0951 (*R =* 0.9998), here *Y* was the peak area, *X* was the quantity (μg) of the sample injected each time, and *R* was the correlation coefficient. The results showed that there was a good linearity for the range of 0.5–6.0 μg in the sample injected.

#### 2.4.3. Precision

The intra- and inter-day precisions are shown in Table 1. The means of the relative standard deviations (RSD) of ustiloxin A content for intra- and inter-day detection at three different levels were less than 0.47% and 0.31%, respectively; and the means of RSD of ustiloxin B content for intra- and inter-day were less than 1.82% and 1.63%, respectively. Both of them were lower than 2.0%, which demonstrates that the method is characterized by good reproducibility.

#### 2.4.3. Accuracy

Accuracy was calculated by means of a standard addition experiment. The results of recovery studies are shown in Table 2. The mean recovery of ustiloxin A was 95.9% (*n* = 9), with a low mean value of RSD of 0.37%, and the mean recovery of ustiloxin B was 92.7% (*n* = 9), with a mean value of RSD of 1.28%. The satisfactory recoveries and low values of the RSDs confirmed the suitability for the analysis of ustiloxins A and B.

#### 2.4.4. Limits of Detection and Quantification

The stock solution of ustiloxins A (**1**) and B (**2**) was diluted to a series of appropriate concentrations with the same solvent, and an aliquot of the diluted solutions was injected into HPLC for analysis. The limits of detection (LOD) and quantification (LOQ) under the present chromatographic conditions were determined at a signa1-to-noise ratio (S/N) of about 3 and 10 with quantities of 0.0315 μg and 0.1212 μg, respectively.

#### 2.4.5. Quantitative Determination of Ustiloxins A and B in Rice False Smut Balls

The contents of ustiloxins A and B in rice false smut balls were determined, and the data are shown in Table 3. The content of ustiloxins A and B in the false smut balls was 0.57 mg/g and 0.38 mg/g, respectively, on a dry weight basis.

## 3. Experimental Section

### 3.1. General

For determination of the main ustiloxins by LC-ESI-MS, liquid chromatography-mass spectrometric analyses were performed using the Agilent 1100 series LC/MSD trap spectrometer (Agilent Technologies, Waldbronn, Germany). MSD, G2446C VL. The HPLC system included a G1311A quaternary pump, a G1313A autosampler, a G1315A detector, a G1379A vacuum degasser with solvent tray and bottles. It was controlled remotely over an Ethernet communication link from a computer using the Hewlett-Packard chemstation system (Agilent Technologies, Waldbronn, Germany). Chromatographic separations were performed at 25 °C and 1 mL/min flow rate using Synergi reversed-phase Hydro-C_18_ column (250 mm × 4.6 mm, 5 μm, Phenomenex, Torrance, CA, USA). The mobile phase, composed of methanol with water containing 0.02% TFA (15:85, *v*/*v*), was eluted at a flow rate of 1.0 mL/min, with UV detection at 220 nm. MS detection was performed on a triple quadrupole analyzer equipped with an ESI source in the positive ion mode. The ESI conditions were as follows: ion source, ESI positive; working mode, SCAN (Scan beginning at 100 *m/z* and ending at 1000 *m/z*); nebulizer gas, N_2_; nebulizer pressure, 35.00 psi; drying gas temperature, 350 °C; drying gas flow rate, 8.0 L/min; capillary HV, 3500 V. Total time of analysis was 30 min.

Ustiloxin content was analyzed by a Prominence LC-20A high-performance liquid chromatography (HPLC) system (Shimadzu, Japan), which consisted of two LC-20AT solvent delivery units, an SIL-20A autosampler, an SPD-M20A photodiode array detector, and a CBM-20Alite system controller. The chromatography column, mobile phase, flow rate and UV detection were the same as those in LC-ESI-MS analysis except a temperature of 30 °C, and a total analysis time of 20 min. The LC solution multi-PDA workstation was employed to acquire and process chromatographic data.

The anion-exchange resin PA308 and the macroporous adsorption resin HP-20 were purchased from Mitsubishi Chemical Holdings, Japan. Sephadex LH-20 was purchased from Pharmacia Biotech, Sweden. The GH gel ODS-AQ was purchased from Daiso, Japan. The Sephadex G-15 was purchased from GE Healthcare, USA. All other chemicals and reagents were of analytical grade.

HR-ESI-MS spectra were measured on Bruker Apex IV FTMS. NMR spectra (^1^H NMR, ^13^C NMR) were recorded on a Bruker Avance DRX-400 NMR spectrometer (^1^H at 400 MHz and ^13^C at 100 MHz). The chemical shifts were expressed in ppm as *δ* values relative to tetramethylsilane (TMS) as an internal standard. Thin layer chromatography (TLC) plates were coated with 0.5-mm layers of silica gel (GF_254_, 300–400 mesh, Qingdao Marine Chemical Company, China). Detection was provided by UV at 254 nm and 365 nm, spraying with 0.2% ninhydrin-acetone (*w*/*v*) reagent.

### 3.2. Material

The rice false smut balls, which were mainly composed of the chlamydospores and mycelia of the rice false smut pathogen (*Villosiclava virens*), were collected from the southwestern part of Shandong Province of China in October 2011. The materials were left to dry in shade at room temperature to a constant weight, and were then stored in sealed plastic bags at −20 °C until required.

### 3.3. Extraction and Fractionation of the Ustiloxins

The rice false smut balls (800 g) were extracted three times with deionized water (2 L each time) at room temperature. The extract was shaken vigorously. The water solution was concentrated under vacuum at 60 °C by a rotary evaporator to afford a concentrated water extract (245 g) which was divided into two parts. The first part (5 g) of the water extract was subjected to anion-exchange resin PA308 chromatography eluted with water at pH 8.0 to water at pH 2.5. The concentrated part, eluted with water at pH 2.5, was used for LC-ESI-MS analysis. The second part (240 g) of water extract was subjected to chromatography over a macroporous adsorption resin HP-20 column (1 L) eluted with distilled water (6 L), then with 30% aqueous ethanol (6 L) to give 28 g of water fraction (containing ustiloxin B) and 7 g of 30% ethanol fraction (containing ustiloxin A), separately.

After that the 30% ethanol fraction was dialyzed with the dialysis tube (pore size, 3500 Da), the concentrated dialysate was chromatographed on an ODS-AQ column using different percentages of methanol containing 0.02% TFA under isocratic conditions. Crude compound **1** was obtained when 5% methanol containing 0.02% TFA was used as an eluent. It was further chromatographed on a Sephadex LH-20 column with MeOH-H_2_O (9:1, *v*/*v*) as an eluent, and then on a Sephadex Q15 column with H_2_O as an eluent to produce pure compound **1** (90 mg). Compound **2** (12 mg) was obtained from the water fraction by using a similar fractionation procedure as compound **1**.

### 3.4. Physicochemical and Spectrometric Data of Ustiloxins A and B

Compound **1** was isolated as a white amorphous powder (MeOH). The molecular formula C_28_H_43_N_5_O_12_S was assigned by HR-ESI-MS, *m/z* 674.26859 [M + H]^+^ (calcd. 674.27017). ^1^H NMR (D_2_O, 400 MHz) δ (ppm): 7.60 (1H, s, H-13), 7.08 (1H, s, H-16), 4.93 (1H, d, *J*_10, 9_ = 10.0 Hz, H-10), 4.84 (1H, s, H-3), 4.35–4.40 (1H, m, H-3′), 4.28 (1H, d, *J*_9, 10_ = 10.0 Hz, H-9), 4.14 (1H, d, *J*_6, 24_ = 10.2 Hz, H-6), 3.99 (1H, dd, *J*_5′, 4′_ = 4.0 Hz, 7.6 Hz, H-5′), 3.77 (2H, s, H-19), 3.33 (1H, dd, *J*_2′, 2′_ = 13.2 Hz, *J*_2′, 3′_ = 9.6 Hz, H-2′), 3.04 (1H, dd, *J*_2′, 2′_ = 13.2 Hz, *J*_2′, 3′_ = 2.8 Hz, H-2′), 2.77 (3H, s, NCH_3_-9), 2.17–2.25 (2H, m, H-22, H-4′), 2.12 (1H, ddd, *J*_4′, 3′_ = 2.8 Hz, *J*_4′, 4′_ = 14.2 Hz, *J*_4′, 5′_ = 7.6 Hz, H-4′), 1.86–1.92 (1H, m, H-24), 1.76 (1H, s, H-21), 1.68–1.73 (1H, m, H-22), 1.09 (3H, t, *J* = 7.2 Hz, H-23), 0.88 (3H, d, *J*_26, 24_ = 7.0 Hz, H-26), 0.78 (3H, d, *J*_25, 24_ = 7.0 Hz, H-25). ^13^C NMR (D_2_O) δ (ppm): 176.0 (C-20), 174.1 (6′-C), 170.7 (C-5), 170.0 (C-17), 166.0 (C-8), 151.9 (C-14), 145.7 (C-15), 136.1 (C-12), 127.7 (C-11), 123.9 (C-16), 113.7 (C-13), 86.9 (C-2), 73.7 (C-10), 66.4 (C-9), 64.5 (2′-C), 63.5 (3′-C), 59.8 (6-C), 59.3 (3-C), 52.5 (5′-C), 43.5 (19-C), 36.4 (4′-C), 32.0 (NCH_3_-C), 31.8 (22-C), 28.4 (24-C), 20.9 (21-C), 18.0 (26-C), 17.7 (25-C), 7.5 (23-C). The data were consistent with the literature [1]. Thus, compound **1** was identified as ustiloxin A.

Compound **2** was isolated as a white amorphous powder (MeOH). The molecular formula C_26_H_39_N_5_O_12_S was assigned by HR-ESI-MS, *m/z* 646.23751 [M + H]^+^ (calcd. 646.23887). ^1^H NMR (D_2_O, 400 MHz) δ (ppm): 7.57 (1H, s, H-13), 7.39 (1H, s, H-16), 4.98 (1H, d, *J*_10, 9_ = 10.0 Hz, H-10), 4.71 (1H, s, H-3), 4.46 (1H, q, *J*_6, 24_ = 7.0 Hz, H-6), 4.33–4.38 (1H, m, H-3′), 4.21 (1H, d, *J*_9, 10_ = 10.0 Hz, H-9), 3.97 (1H, dd, *J*_5′, 4′_ = 4.0 Hz, 8.0 Hz, H-5′), 3.81 (1H, d, *J*_19, 19_ = 17.0 Hz, H-19), 3.75 (1H, d, *J*_19, 19_ = 17.0), 3.37 (1H, dd, *J*_2′, 2′_ = 13.4 Hz, *J*_2′, 3′_ = 10.0 Hz, H-2′), 3.03 (1H, dd, *J*_2′, 2′_ = 13.4 Hz, *J*_2′, 3′_ = 2.4 Hz, H-2′), 2.76 (3H, s, NCH_3_-9), 2.04–2.19 (3H, m, H_2_-4′, H-22), 1.74 (3H, s, H-21), 1.65–1.70 (1H, m, H-22), 1.19 (3H, d, *J*_24, 6_ = 7.0 Hz, H-24), 0.97 (3H, t, *J*_23, 22_ = 7.2 Hz, 7.2 Hz, H-23). ^13^C NMR (D_2_O) δ (ppm): 176.2 (C-20), 174.1(6′-C), 171.8 (C-5), 169.8 (C-17), 165.6 (C-8), 152.0 (C-14), 145.6 (C-15), 136.5 (C-12), 127.8 (C-11), 123.9 (C-16), 113.7 (C-13), 87.0 (C-2), 73.3 (C-10), 66.1 (C-9), 64.5 (2′-C), 63.5 (3′-C), 59.6 (3-C), 52.4 (5′-C), 49.4 (6-C), 43.5 (19-C), 36.4 (4′-C), 31.7 (NCH_3_-C), 30.9 (22-C), 21.6 (21-C), 15.2 (24-C), 7.7 (23-C). The data were consistent with the literature [1]. Therefore, compound **2** was identified as ustiloxin B.

### 3.5. HPLC Analysis of Ustiloxins A and B

#### 3.5.1. Preparation of the Ustiloxin Standard Solutions

An amount of 1 mg of ustiloxin A or ustiloxin B was accurately weighed. They were mixed and dissolved in 1 mL of methanol-water (15:85, *v*/*v*) as the mother solution at 1000 μg/mL for each ustiloxin. The mother solution was further diluted into 150 μg/mL, 125 μg/mL, 100 μg/mL, 62.5 μg/mL, 50 μg/mL, 31.25 μg/mL, 25 μg/mL and 12.5 μg/mL samples with methanol-water (15:85, *v*/*v*), and the solutions were kept at 4 °C.

#### 3.5.2. Preparation of Sample Solutions

Based on the physicochemical properties of the ustiloxins, each powdered sample of rice false smut balls (200 mg) was extracted with deionized water (3 × 6 mL) in an ultrasonic bath at room temperature. The extraction time for each extraction was 4 h. The water extract was then concentrated under vacuum at 60 °C by a rotary evaporator to dryness and dissolved in 5 mL of methanol-water (15:85, *v*/*v*). It was then filtered through a filter (pore size, 0.22 μm) before analysis.

#### 3.5.3. Method Validation

The HPLC-UV method was completely validated according to the procedures described in ICH guidelines Q2 (R1) for the validation of analytical methods [12,13].

The intra-day precision was examined by analysis of authentic ustiloxins A and B at levels 150 μg/mL, 50 μg/mL and 12.5 μg/mL, which were prepared and analyzed on the same day. Each level of the ustiloxins A and B was detected for five repetitions (*n* = 5). Similarly, the inter-day precision was assessed by analyzing the aforementioned three levels of ustiloxins A and B on three separate days (*n* = 9). The peak areas of the ustiloxins A and B and the relative standard deviations (RSDs) were calculated [14].

The accuracy of the method was determined by analyzing the mixtures which were obtained by adding known amounts of standard ustiloxins A and B to the rice false smut balls (200 mg) in which the contents of ustiloxins A and B were known. The added amounts of authentic ustiloxins A and B were at low, medium, and high levels. The samples were prepared in triplicate, and the recovery percentages and RSDs were calculated.

The limit of detection (LOD) and limit of quantification (LOQ) were based on signal-to-noise approach. Determination of the signal-to-noise ratio is performed by comparing measured signals from samples with known low concentrations of analyte with those of blank samples and by establishing the minimum concentration at which the analyte can be reliably detected or quantified [15]. A signal-to-noise ratio between 3:1 and 2:1 is generally considered acceptable for estimating the detection limit, and a typical signal-to-noise ratio is 10:1 for the quantification limit. Both LOD and LOQ were expressed by the quantity (μg) of ustiloxins A and B.

#### 3.5.4. Quantitative Determination of Ustiloxins A and B in Rice False Smut Balls

The contents of ustiloxins A and B in rice false smut balls were determined by HPLC-UV. The solutions of the crude extracts were prepared as described in Section 3.5.2. The data were presented as an average of six replicates for each sample. The experiments were repeated three times. The relative standard deviations (RSDs) were also calculated.

## 4. Conclusions

In this study ustiloxins A and B as the two main mycotoxins were determined conveniently by LC-ESI-MS in the water extract from rice false smut balls. Both ustiloxins A and B in rice false smut balls were also quantitatively analyzed by HPLC-UV. To the best of our knowledge, this is the first report on the determination and analysis of ustiloxins A and B in rice false smut balls by LC-ESI-MS and HPLC methods simultaneously. The LC-ESI-MS method should be a practical way for rapid determination and analysis of the main ustiloxins in test samples. Isolation and structural identification of other ustiloxins (*i.e*., ustiloxins C and D) from rice false smut balls are in progress. It is worth to mention that ustiloxins have been considered as the antimitotic compounds that may exhibit a broad range of biological activities and can be used for medicinal and agrochemical purposes, acting as anticancer, antifungal, and anthelmintic agents [3,16]. Moreover, ustiloxins as tubulin-binding compounds are valuable for understanding basic mechanisms involved in the dynamics of the microtubule network [17–19].

## Supplementary Materials



## Figures and Tables

**Table 1:** Precision and relative standard deviations (RSDs) of ustiloxin A and B determinations by HPLC.

Compound	Intra-day (*n* = 5)		Inter-day (*n* = 9)	

	Concentration (μg/mL)	RSD (%)	Concentration (μg/mL)	RSD (%)
Ustiloxin A	12.5	0.47	12.5	0.24
	50.0	0.14	50.0	0.27
	150.0	0.07	150.0	0.31

Ustiloxin B	12.5	1.82	12.5	1.63
	50.0	0.29	50.0	0.13
	150.0	1.43	150.0	1.45

**Table 2:** Recoveries and RSDs of ustiloxins A and B in rice false smut balls.

Compound	Added concentration (μg/mL)	Recovery yield (%)	RSD (%)
Ustiloxin A	16.0	97.4	0.49
	32.0	96.9	0.26
	64.0	93.4	1.73

Ustiloxin B	16.0	90.0	0.65
	32.0	91.4	1.85
	64.0	96.5	1.34

**Table 3:** Contents and RSDs of ustiloxins A and B in rice false smut balls.

Compound	Average (mg/g, *n* = 6)	RSD (%)
Ustiloxin A	0.57 ± 0.0078	1.38
Ustiloxin B	0.38 ± 0.0042	1.11
