# Supplementary material for: Determination and Analysis of Ustiloxins A and B by LC-ESI-MS and HPLC in False Smut Balls of Rice
Source: Int J Mol Sci. 2012 Sep 10;13(9):11275–87. doi: 10.3390/ijms130911275 (PMC3472744; doi:10.3390/ijms130911275)

Supplementary Materials

Figure S1. HR-ESI-MS spectrum of compound **1** (ustiloxin A).

Analysis Info

Analysis Name	12051040_20120528_000002.d	Acquisition Date	5/28/2012 11:09:23 AM
Sample	Uvf-6	Instrument	Bruker Apex IV FTMS
Comment	ESI Positive	Operator	Peking University

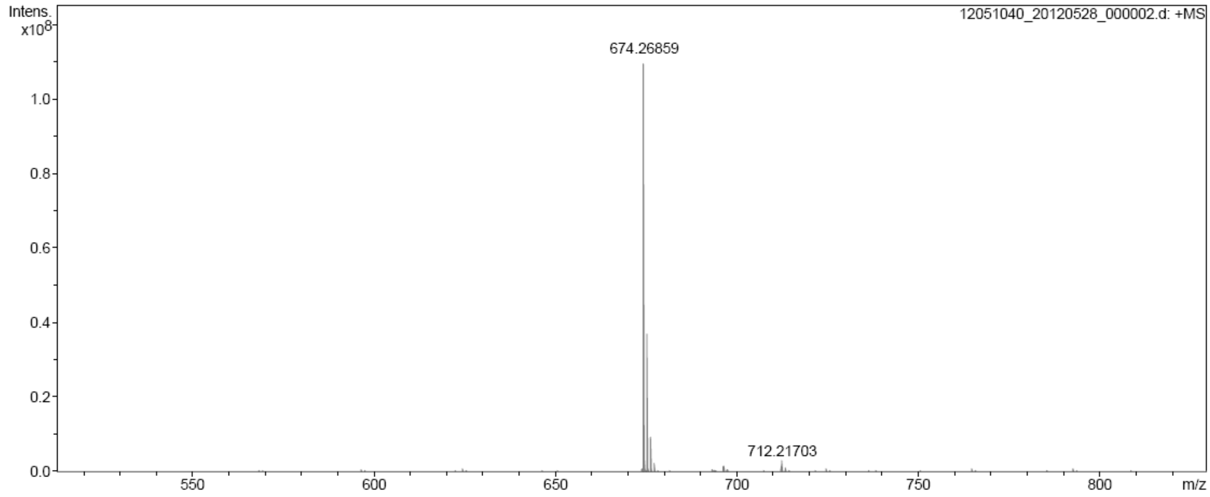

Meas. m/z	#	Formula	Score	m/z	err [mDa]	err [ppm]	mSigma	rdb	e⁻ Conf	N-Rule
674.26859	1	C 28 H 44 N 5 O 12 S	100.00	674.27017	1.6	2.3	21.0	9.5	even	ok

Figure S2. ^{1}H NMR spectrum of compound **1** (ustiloxin A).

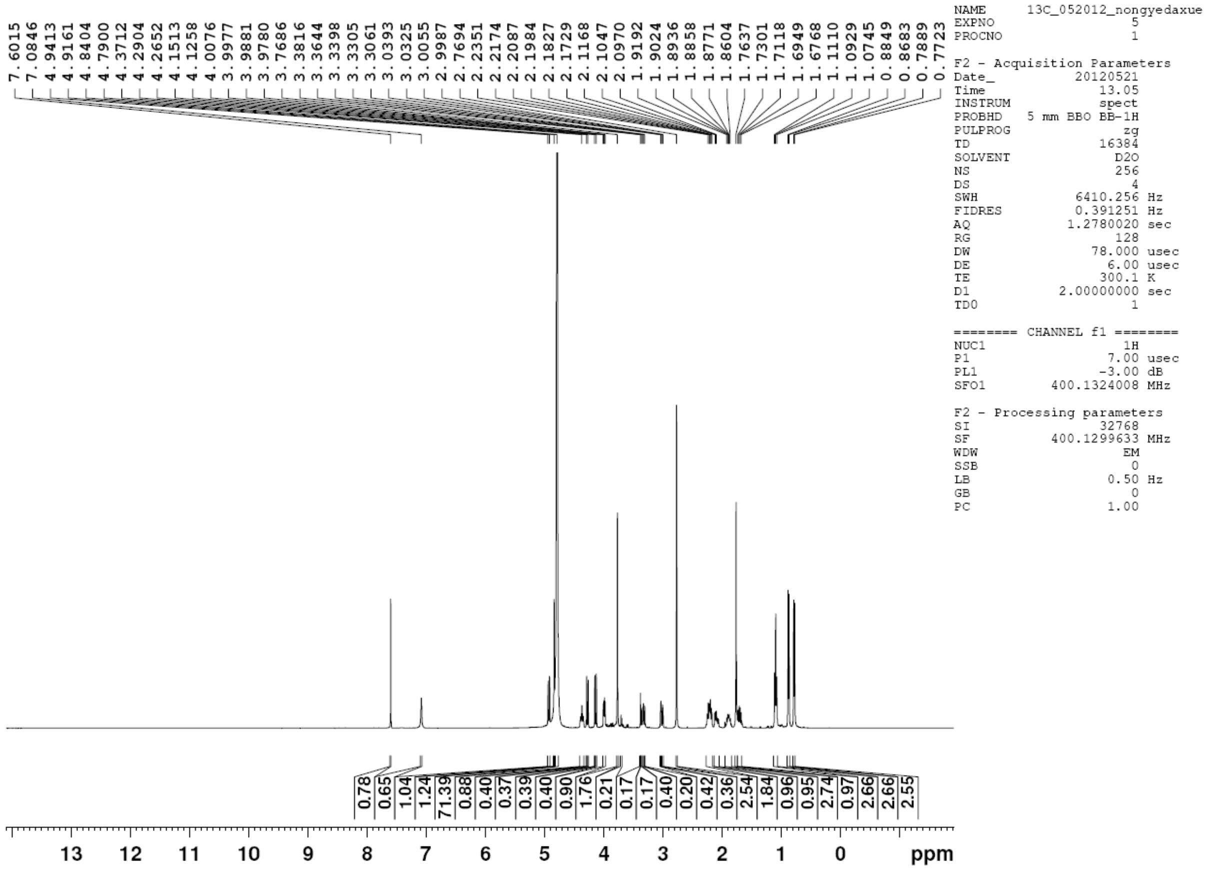

Figure S3. ^{13}C NMR spectrum of compound **1** (ustiloxin A).

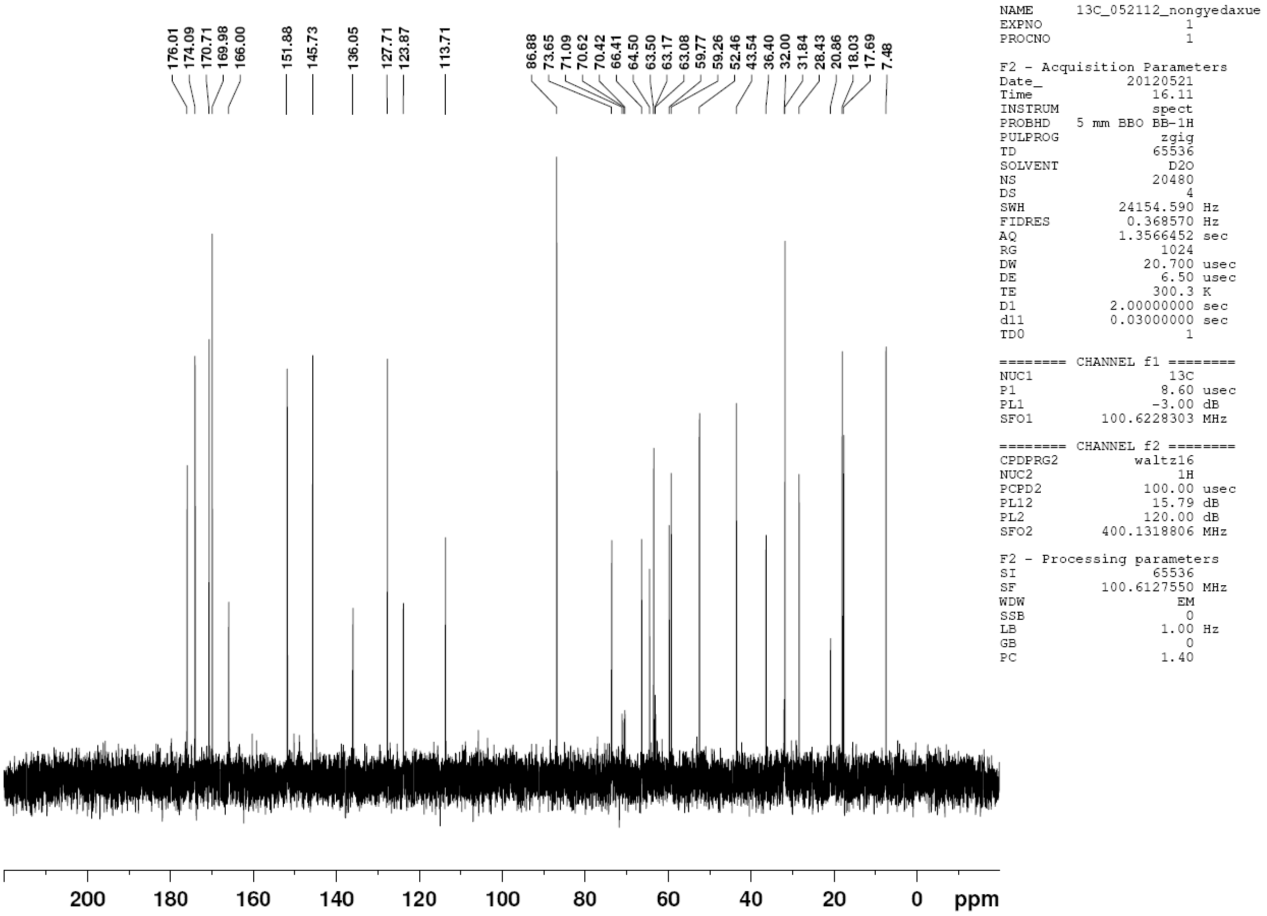

Figure S4. HR-ESI-MS spectrum of compound **2** (ustiloxin B).

Analysis Info

Analysis Name	12051039_20120528_000001.d	Acquisition Date	5/28/2012 11:05:37 AM
Sample	Uvf-5	Instrument	Bruker Apex IV FTMS
Comment	ESI Positive	Operator	Peking University

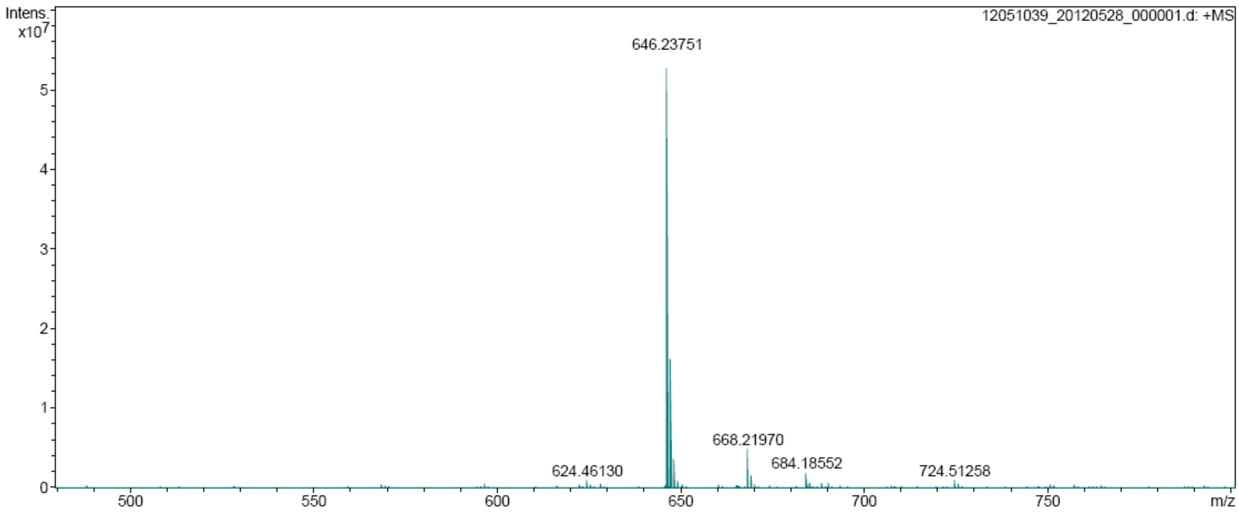

Meas. m/z	#	Formula	Score	m/z	err [mDa]	err [ppm]	mSigma	rdb	e⁻ Conf	N-Rule
646.23751	1	C 26 H 40 N 5 O 12 S	100.00	646.23887	1.4	2.1	27.7	9.5	even	ok

Figure S5. ^{1}H NMR spectrum of compound **2** (ustiloxin B).

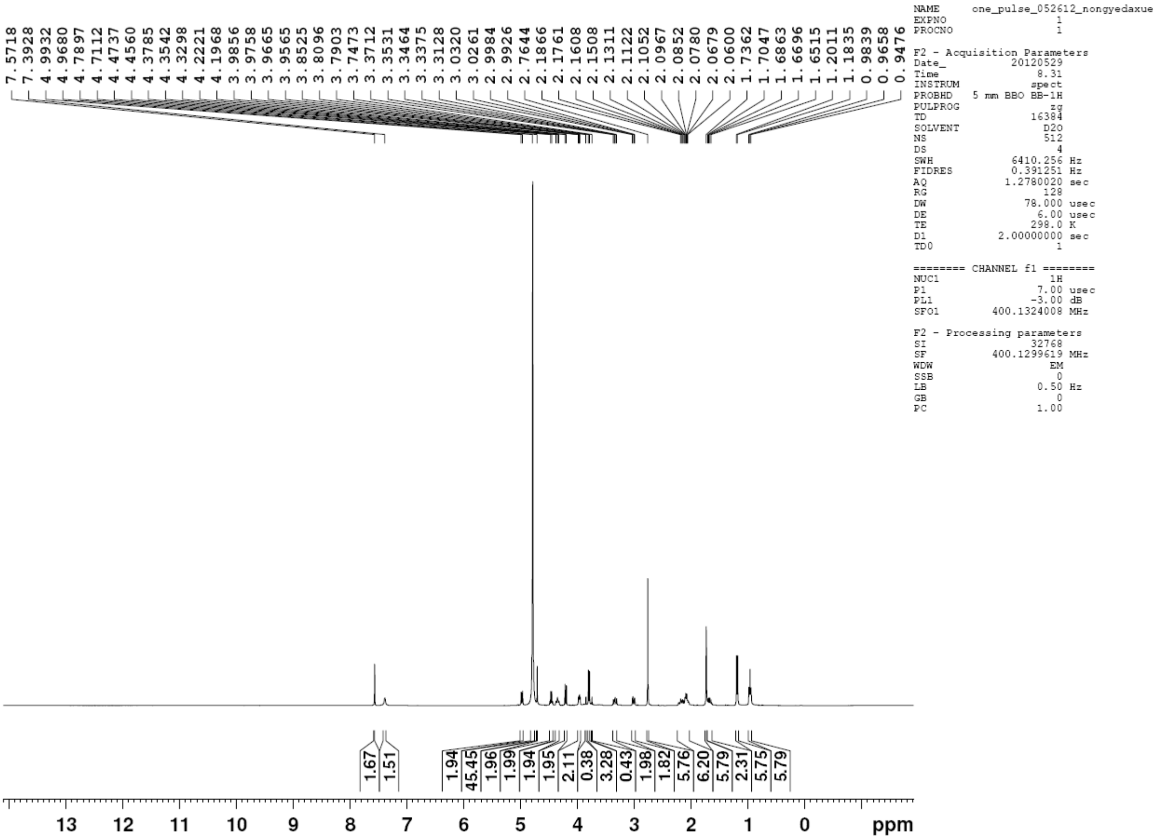

Figure S6. ^{13}C NMR spectrum of compound **2** (ustiloxin B).

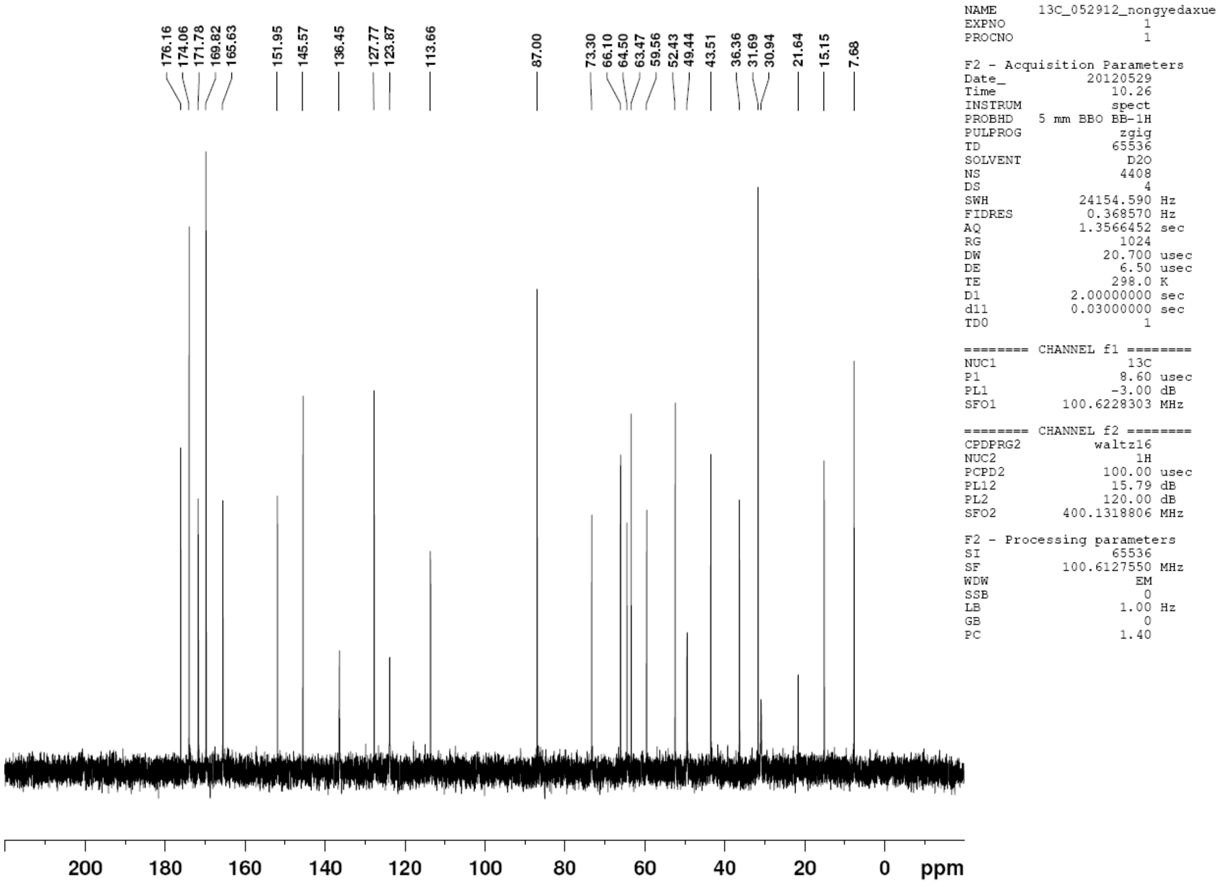

Supplement: Supplementary file 1 [file ijms-13-11275-s001.pdf]
